# Identification of Five Tumor Antigens for Development and Two Immune Subtypes for Personalized Medicine of mRNA Vaccines in Papillary Renal Cell Carcinoma

**DOI:** 10.3390/jpm13020359

**Published:** 2023-02-18

**Authors:** Jianpei Hu, Zhongze Yuan, Yifen Jiang, Zengnan Mo

**Affiliations:** 1 Center for Genomic and Personalized Medicine, Guangxi Key Laboratory for Genomic and Personalized Medicine, Guangxi Collaborative Innovation Center for Genomic and Personalized Medicine, Guangxi Medical University, Nanning 530021, China; 2 Institute of Urology and Nephrology, First Affiliated Hospital of Guangxi Medical University, Nanning 530021, China; 3 Department of Urology, The First Affiliated Hospital of Guangxi Medical University, Nanning 530021, China; 4 Department of Medical Record Management Center, The People’s Hospital of Yubei District of Chongqing City, Chongqing 401120, China

**Keywords:** papillary renal cell carcinoma, tumor antigens, mRNA vaccine, immune landscape, personalized medicine

## Abstract

Increasing evidence has revealed the promise of mRNA-type cancer vaccines as a new direction for cancer immune treatment in several solid tumors, however, its application in papillary renal cell carcinoma (PRCC) remains unclear. The purpose of this study was to identify potential tumor antigens and robust immune subtypes for the development and appropriate use of anti-PRCC mRNA vaccines, respectively. Raw sequencing data and clinical information of PRCC patients were downloaded from The Cancer Genome Atlas (TCGA) database. The cBioPortal was utilized for the visualization and comparison of genetic alterations. The TIMER was used to assess the correlation between preliminary tumor antigens and the abundance of infiltrated antigen presenting cells (APCs). Immune subtypes were determined by the consensus clustering algorithm, and clinical and molecular discrepancies were further explored for a deeper understanding of immune subtypes. Five tumor antigens, including ALOX15B, HS3ST2, PIGR, ZMYND15 and LIMK1, were identified for PRCC, which were correlated with patients’ prognoses and infiltration levels of APCs. Two immune subtypes (IS1 and IS2) were disclosed with obviously distinct clinical and molecular characteristics. Compared with IS2, IS1 exhibited a significantly immune-suppressive phenotype, which largely weakened the efficacy of the mRNA vaccine. Overall, our study provides some insights for the design of anti-PRCC mRNA vaccines and, more importantly, the selection of suitable patients to be vaccinated.

## 1. Introduction

Renal cell carcinoma (RCC), histologically stemming from the tubular epithelial cell layer, represents the most frequently occurring kidney neoplasms encountered by urologists, which surpasses 90% of all kidney malignancies [1]. It is estimated that, in the year 2020, there were about 430 thousand newly-diagnosed RCC cases all over the world, seriously jeopardizing the well-being of humans and becoming a burden on our societies [2]. Papillary renal cell carcinoma (PRCC) is the most common subtype of the non-clear RCC, accounting for nearly 20% of all subtypes with distinctly different genetic, morphological and clinical characteristics in comparison with the clear cell RCC [3]. For early-stage patients suffering from PRCC, surgical extirpation is always the first choice, with the maximal possibility to be cured and a favorable prognosis; although a portion of PRCC patients unfortunately progress to metastatic disease with a dismal prognosis [4]. Building on a deep understanding of the oncogenesis and progression mechanisms of PRCC, molecular targeting therapies such as sunitinib and crizotinib, aimed at vascular endothelial growth factor (VEGF) and the c-MET pathway, respectively, together with subsequent immune checkpoint blockers, have shown the capacity to retard the aggression of malignant cells; nonetheless, the efficacy of these remedies is frequently limited or impermanent, for example, the response rate to therapeutic drugs (encompassing sunitinib and sorafenib) is only 9.2%, and results from a phase II trial show that merely 4 in 23 PRCC patients treated with crizotinib achieved partial responses [5,6,7]. Hence, with the purpose of refining the prognosis, it is of tremendous necessity to search for other therapies for patients with PRCC.

In recent years, accumulating attention has been paid by scholars worldwide, to the development of the cancer vaccine, which attempts to enhance the patients’ immunity against cancerous cells [8]. The discovery of tumor associated antigens (TAAs) represents one of the major challenges in the design of a vaccine, and several studies have introduced new ideas to the identification of TAAs [9,10,11]. In the family of cancer vaccines, compared to other members, messenger RNA (mRNA) vaccines possess some unique advantages such as: synchronously eliciting the humoral and cell immunity, without a risk of integration into the nuclear genome and encoding more epitopes to be presented by antigen presenting cells (APCs) [12]. Nowadays, the effectiveness and safety of mRNA vaccines have been fully affirmed in the fight against COVID-19, and this epidemic objectively facilitates the pace of developing therapeutic mRNA vaccines including those for cancers [13]. Both CV9103 (encoding four tumor antigens) and CV9201 (encoding five tumor antigens) are mRNA vaccines that treat patients with advanced prostate cancer and non-small lung cell cancer, respectively, and preliminary results have revealed they are well tolerated as well as having significant immunogenicity [14,15]. Moreover, several other clinical trials are underway to investigate the performance and safety of mRNA vaccines in other types of malignancies encompassing melanoma, head and neck squamous carcinoma and colorectal cancer [16]. As a form of immunotherapy, the outcome of mRNA vaccines is inevitably affected by the status of the tumor immune microenvironment (TIME), especially the infiltration degree and function of CD8^+^ T cells [17]. Nevertheless, as far as we know, there are currently no published articles with respect to the development of mRNA vaccines for patients with PRCC.

The goals of this study were the identification of tumor antigens for the design of mRNA-based vaccines against PRCC and the precision medicine of this remedy assisted by characterizing the immune subtypes of patients. Altogether, we provide some new ideas about the development of mRNA vaccines opposing PRCC, as well as the appropriate selection of patients with heterogeneous TIME to be vaccinated.

## 2. Materials and Methods

### 2.1. Obtaining and Processing Public Data 

Raw bulk sequencing data (counts format) of PRCC deposited in the comprehensive TCGA project were downloaded via the Xena Platform [18] (https://xena.ucsc.edu/, accessed on 7 August 2022) and matched to the clinical and survival information of each patient. Moreover, openly free data in the “maf” format, accessible at the Genomic Data Commons Data Portal (https://portal.gdc.cancer.gov/, accessed on 7 August 2022), were deeply analyzed by means of the “maftools” (an R package) for the disclosure of mutated genes, mutation frequency, tumor mutation burden (TMB), and expression correlations among mutated genes of interest.

### 2.2. cBioPortal Analysis

The online cBioPortal tool (version 5.0.2, http://www.cbioportal.org/, accessed on 7 August 2022) was inaugurated for worldwide researchers especially those interested in cancer genomics, gathering manifold data mainly from tissue samples (TCGA, International Cancer Genome Consortium) and malignant cells (Cancer Cell Line Encyclopedia), and others [19]. Herein, relying on a total of 293 samples from the TCGA cohort, the genome alteration status of PRCC was entirely detected and visualized by right of the cBioPortal for the dissection of potential tumor antigens.

### 2.3. GEPIA Analysis

The Gene Expression Profiling Interactive Analysis (GEPIA, available at http://gepia2.cancer-pku.cn/, accessed on 7 August 2022) incorporated deep-sequencing data from samples (dominated by cancerous specimens) in the TCGA database along with normal samples sequenced by the Genotype-Tissue Expression (GTEx) project as a complement, and it was diffusely used to look into differential genes resulting from malignant transformation in as many as 33 types of malignancies [20]. Genes that were over-accumulated (Log2 fold change > 1) in PRCC tumor tissues rather than in normal renal tissues, concurrent with statistical significance (q-value < 0.01), were obtained by the LIMMA method.

### 2.4. Identification of Genes Associated with PRCC Patients’ Prognoses

Samples from patients whose overall survival time more than 30 days were remaining, and genes that were expressed in less than half of samples were excluded for subsequent analyses. Firstly, raw counts data were converted to counts per million (CPM) values through the “cpm” function in the “edgeR” package. Then, on the strength of the median expression level of a given gene, patients were split into two groups: the high-expressed group and the low-expressed group. Lastly, two professional R packages “survival” and “survminer” were used to perform survival analyses; the Kaplan–Meier method along with log rank testing were united to ascertain whether notable differences in overall survival (OS) time were observed between groups.

### 2.5. TIMER Analysis

In late 2017, the Tumor IMmune Estimation Resource (TIMER) [21], an all-round web server characterized by multiple functional modules (https://cistrome.shinyapps.io/timer/, accessed on 20 August 2022) was created for researchers to overcome numerous difficulties in analyzing immune infiltrates in malignant tumor tissues; more specifically, by means of the TIMER algorithm, the richness of six vital types of immune cells including neutrophils, B cells, dendritic cells, CD4^+^ T cells, CD8^+^ T cells and macrophages was estimated across various solid tumors. Correlations between expression levels of genes and infiltrative degrees of three kinds of immune cells with antigen-presenting capabilities, namely macrophages, dendritic cells and B cells, were assessed through the gene module. Subsequent to the correction of the influence of tumor purity, the partial Spearman’s rho value coupled with its statistical *p* value were calculated and outputted for visualizing these latent associations.

### 2.6. Discovery of the Immune Subtypes

A list of ~4700 immunologically related genes was acquired from the InnateDB database (https://www.innatedb.ca/, accessed on 25 August 2022) and a total of 4677 genes were retained after removing duplicated genes. Expression of genes possessing immune system-related functions in TCGA-PRCC samples were taken from the original matrix to serve as input data for the “ConsensusClusterPlus” R package [22], aimed at revealing underlying immune subtypes in these patients. To accomplish this clustering, the partition around medoids (a cluster algorithm) in conjunction with “1-Pearson correlation” distance were chose to be actual arguments; the number of subsamples, the upper limit of resulting clusters and the proportion of resampling were set as 500, 6 and 80%, respectively. The optimal number of clustering was determined by simultaneously considering results from the consensus matrix, the consensus cumulative distribution function (CDF), as well as the delta area. Subsequently, principal component analysis was used to evaluate whether the samples from one immune subtype could be properly differentiated from other subtypes. Next, the “survival” R package was applied to compare the overall survival time of patients that were grouped into discrete subtypes. Eventually, intrinsic signatures of immune subtypes in terms of clinical parameters and tumor mutation burden were disclosed for deeper understanding.

### 2.7. Degree of Immune Cell Infiltration Analysis

In the first place, the ESTIMATE method deduced proportions of stromal and immune components from gene expression signatures in PRCC patients’ tumor samples which were exported as the stromal score and immune score, respectively [23]. Furthermore, each type of immune cell was unique, with its own characteristic gene expression pattern; based on this concept, the single sample gene set enrichment analysis (ssGSEA) was performed to explore the abundance of a total of twenty-eight types of immune cells, assisted by the “GSVA” package [24]. Lastly, the “CIBERSORT” algorithm inferring the fraction of twenty-two kinds of immune cells in tumor samples was also adopted to delineate the immune cell landscape from the complicated tumor microenvironment [25].

### 2.8. Differential Analysis of ICD Modulators and ICPs

Both immunogenic cell death (ICD) modulators and immune checkpoints (ICPs) were important in adjusting the degree of immune activation using different mechanisms, and these molecules were obtained from previously published papers [26,27]. Afterwards, the expression levels of these genes in PRCC samples were extracted and compared by immune subgroups.

### 2.9. Gene Co-Expression Network Analysis

The “WGCNA” package in R software (version 4.1.0) [28] was utilized to uncover co-expression modules of genes, as well as correlations between modules and immune phenotypes. Considering that non-variant and low-variant genes were always representative of noise, these genes were firstly filtered out; outliers measured by the sample tree were also eliminated from subsequent analyses. Thereafter, the “pickSoftThreshold” function calculated the optimal soft threshold, serving as a prerequisite of the “blockwiseModules” function to construct a co-expression matrix in one step. Subsequently, modules were visualized with different colors, and links between modules and immuno-subtypes were questioned. Finally, with the aid of the “clusterProfiler” package [29], gene ontology (GO) analysis was used to annotate genes in the module that were most correlated with immune subtypes.

### 2.10. Anticancer Drug Sensitivity Analysis

The impacts of genomic alterations in cancerous cells on patients’ responses to multifarious anti-cancer drugs indeed existed but were ill-defined. The genomics of drug sensitivity in cancer (GDSC) database was developed to fill this gap in 2013 [30]. This database was constituted mainly of two data sets: the gene expression pattern of diverse cancer cells without disturbance and sensitivities of these cells to hundreds of commonly used compounds quantified by the half maximal inhibitory concentration (IC50). On the basis of a ridge regression model, the R package “oncoPredict” [31], using data from the GDSC as a training set, could predict the sensitivity of PRCC patients to anticarcinogens, and drug-sensitivity variations in individuals with different immune subtypes were investigated further.

## 3. Results

### 3.1. Identification of Potential Tumor Antigens of PRCC

**Figure 1:**
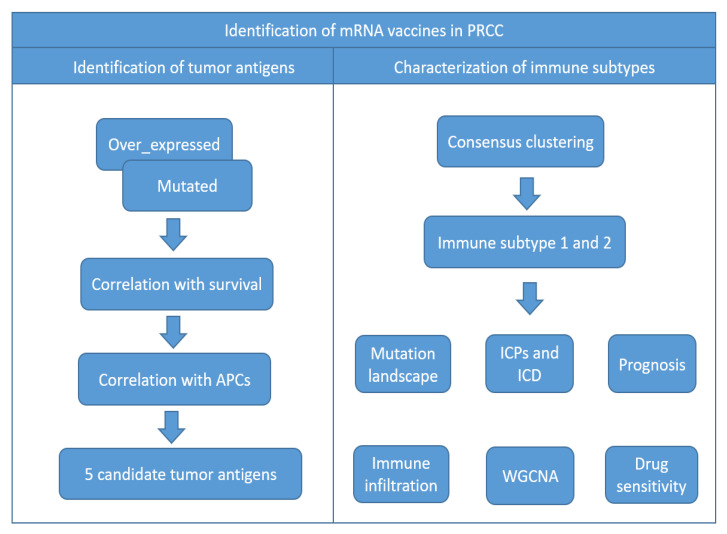
The work flow of the study.

**Figure 2:**
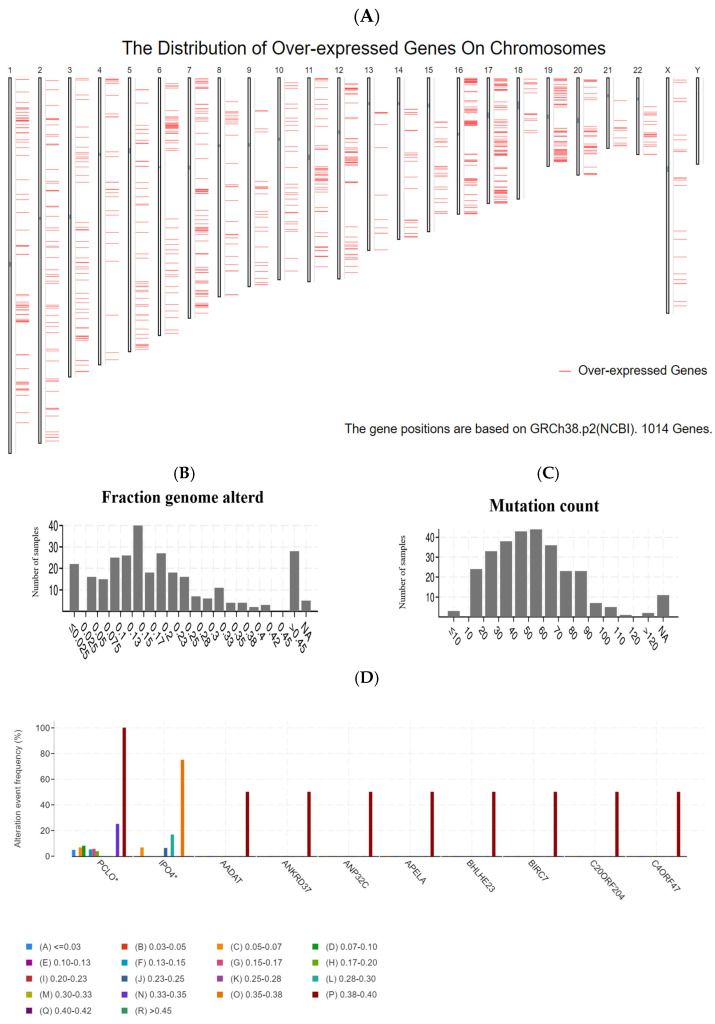

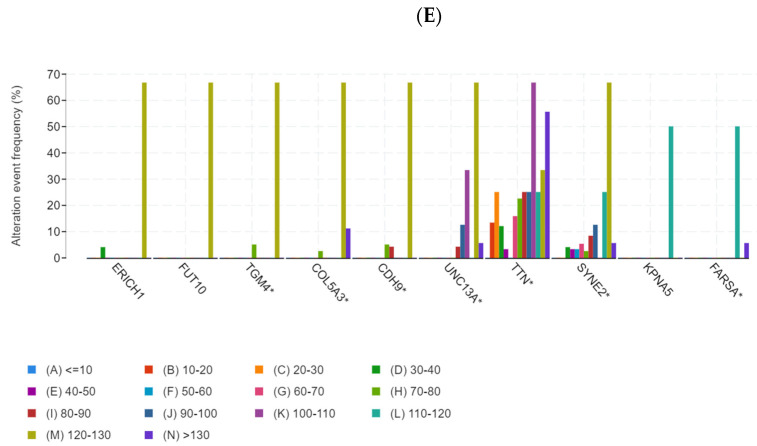
Identification of potential tumor antigens of PRCC. (**A**) Upregulated genes in PRCC mapped onto human chromosomes. (**B**,**C**) Number of samples in the altered fraction genome group and mutation count group, respectively. (**D**) Top ten alteration event frequencies in the fraction genome altered group. (**E**) Top ten alteration event frequencies in the mutation count group.

A flowchart of the study is shown in Figure 1. In the quest for potential tumor antigens of PRCC, the abnormally over-accumulated genes were interrogated and a total of 1014 up-regulated genes were screened out, which held the likelihood of encoding TAAs (Figure 2A). Adding up to 8315 mutated genes (also prone to generate TAAs) allowed subsequent identification, by virtue of profiling the altered genome fraction (Figure 2B) in conjunction with mutation counts (Figure 2C) in each sample. As shown in Figure 2D, the top ten frequently mutated genes, within the fraction of the genome altered group, were piccolo presynaptic cytomatrix protein (PCLO), importin 4 (IPO4), aminoadipate aminotransferase (AADAT), ankyrin repeat domain 37 (ANKRD37), acidic nuclear phosphoprotein 32 family member C (ANP32C), apelin receptor early endogenous ligand (APELA), basic helix-loop-helix family member e23 (BHLHE23), baculoviral IAP repeat containing 7 (BIRC7), chromosome 20 open reading frame 204 (C20ORF204), chromosome 4 open reading frame 47 (C4ORF47). In addition, within the mutation count group (Figure 2E), the top ten genes were as follows: glutamate rich 1 (ERICH1), fucosyltransferase 10 (FUT10), transglutaminase 4 (TGM4), collagen type V alpha 3 chain (COL5A3), cadherin 9 (CDH9), unc-13 homolog A (UNC13A), titin (TTN), spectrin repeat containing nuclear envelope protein 2 (SYNE2), karyopherin subunit alpha 5 (KPNA5), phenylalanyl-tRNA synthetase subunit alpha (FARSA). In aggregate, 333 genes were ascertained by the intersection of mutated and overexpressed genes.

### 3.2. Identification of Tumor Antigens Associated with Antigen Presenting Cells and Patients’ Prognoses

**Figure 3:**
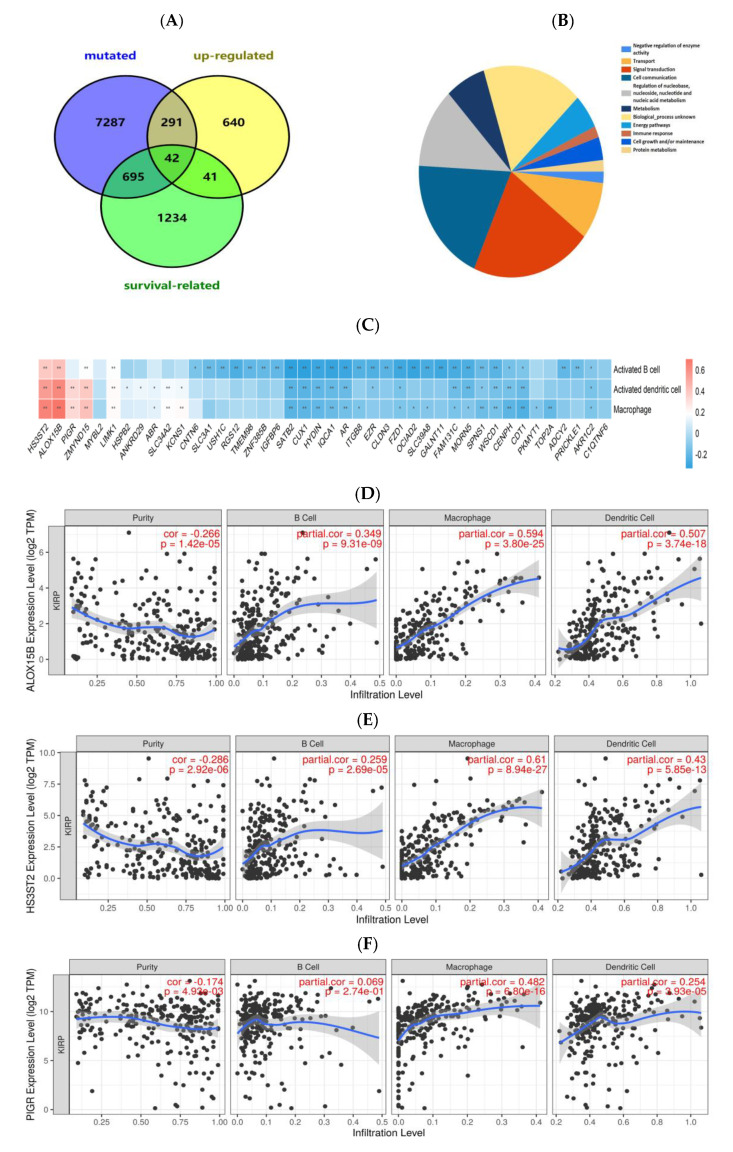

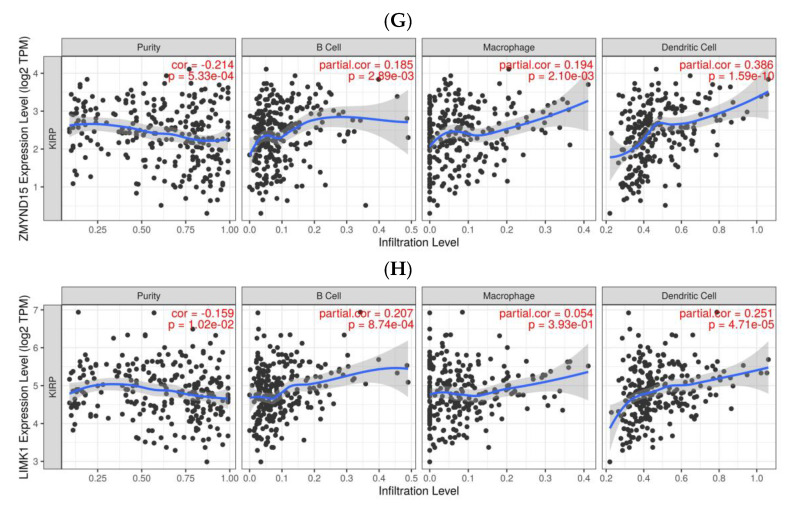
The associations between five potential PRCC antigens and three types of APCs. (**A**) The number of separate and common genes among mutated, upregulated and survival-related groups. (**B**) Pie chart from Funrich [32] revealing the major biological processes these common genes participate in. (**C**)The expression associations between intersected genes and APCs based on the ssGSEA method. (**D**–**H**) The expression correlations between ALOX15B (**D**), HS3ST2 (**E**), PIGR (**F**), ZMYND15 (**G**), LIMK1 (**H**) and infiltration degrees of APCs were quantified with the TIMER database.

**Figure 4:**
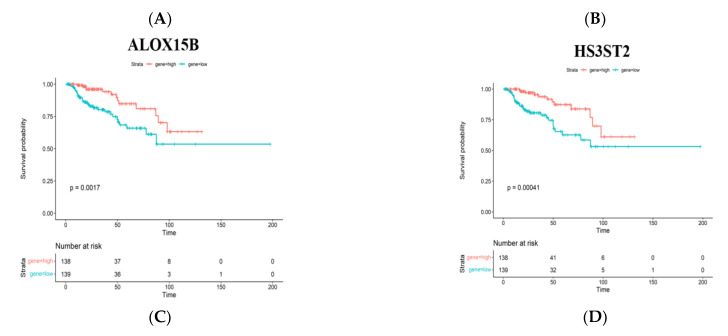

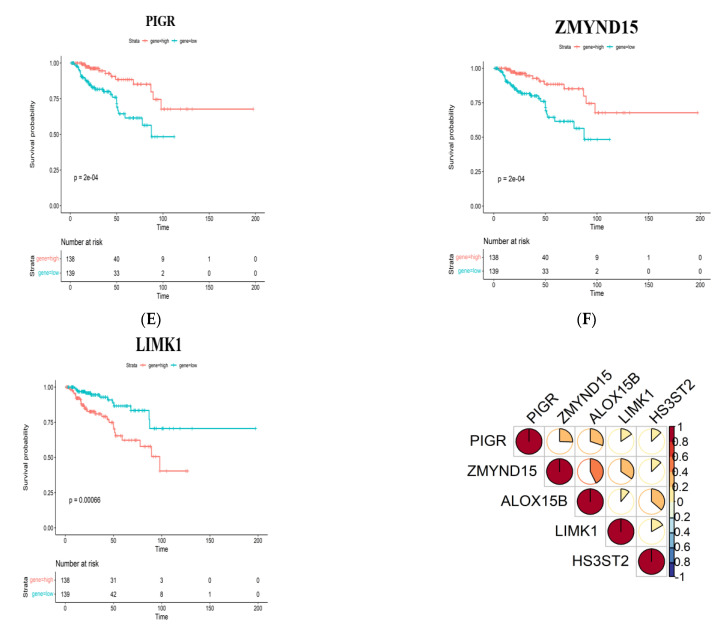
Identification of tumor antigens associated with PRCC prognosis. (**A**–**E**) Kaplan–Meier curves showing the overall survival probability of PRCC patients in groups with different expressions of ALOX15B (**A**), HS3ST2 (**B**), PIGR (**C**), ZMYND15 (**D**) and LIMK1 (**E**). (**F**) The expression correlations among the five genes.

The prognostic value of these mutated and amplified genes was further analyzed by sieving out prognosis-pertinent tumor antigens that could be considered as candidates for the design of mRNA vaccines. In light of the Kaplan–Meier (KM) analysis, a total of 2012 OS-related genes remained, and 42 common genes were identified which participated in subsequent analyses (Figure 3A), which were mainly involved in signal transduction, cell communication, metabolism, and immune response (Figure 3B). Next, from the perspective of cancer vaccine development, expression correlations between these genes with macrophages, dendritic cells and B cells were thoroughly investigated, indicating that only 5 out of 42 genes showed significantly positive correlations (Figure 3C–H). The lower expressions of arachidonate 15-lipoxygenase type B (ALOX15B), heparan sulfate-glucosamine 3-sulfotransferase 2 (HS3ST2), polymeric immunoglobulin receptor (PIGR), zinc finger MYND-type containing 15 (ZMYND15) and higher expression of LIM domain kinase 1 (LIMK1) were relevant to prominently deteriorated OS time of patients with PRCC (Figure 4A–E). The expression correlations between the five genes were displayed (Figure 4F). Taken together, five tumor antigens (ALOX15B, HS3ST2, PIGR, ZMYND15, and LIMK1), with potentially provocative effects on immunological functions, were identified and considered as eligible candidates for anti-PRCC mRNA vaccine development.

### 3.3. Identification of Immune Subtypes of PRCC

**Figure 5:**
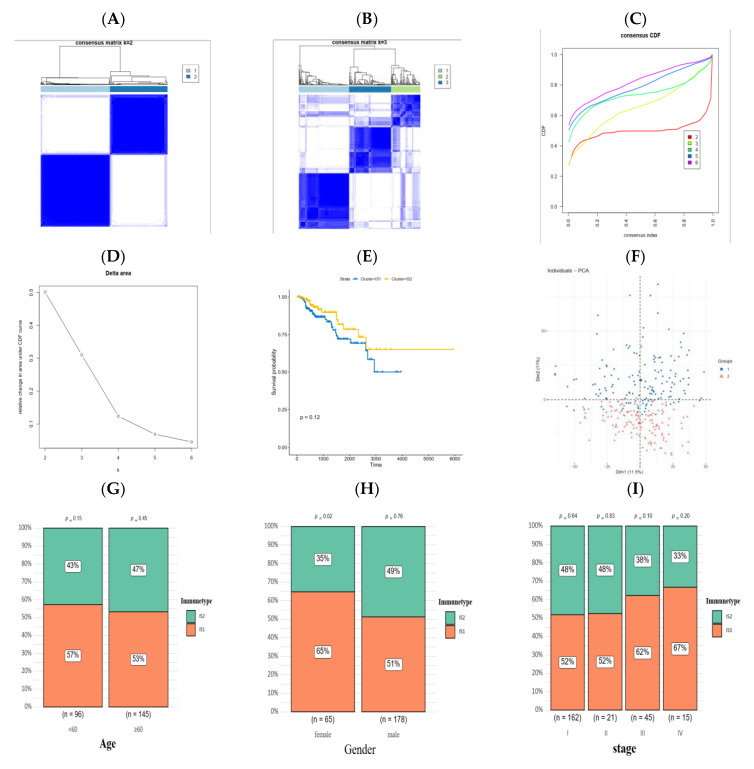
Identification of immune subtypes in individuals with PRCC. (**A**) Consensus clustering matrix of PRCC samples when k = 2. (**B**) Consensus clustering matrix of PRCC samples when k = 3. (**C**) Consensus clustering CDF when k was in the range of 2 to 6. (**D**) Relative area under CDF curve changed when k was in the range of 2 to 6. (**E**) Survival analysis between OS and two groups. (**F**) The prognosis discrepancy of patients in the two clusters. (**G**–**I**) Different proportions of immune subtypes in age, gender, and stage groups.

The heterogenous immune status of the tumor microenvironment can be disentangled through exploring the expression pattern of immunological related genes, and the immune subtype significantly impacts the efficacy of the mRNA vaccine, hence it is a useful indicator for the proper selection of PRCC patients to receive a vaccine. The expression of 4723 immunologically relevant genes, in PRCC samples, were extracted from the matrix for the construction of consensus clustering. As shown in Figure 5A,B, when k = 2 the white part in the consensus matrix was clearly clean without blue additions, and two diverse immune subtypes were obtained after combining with the results from the consensus distribution function (Figure 5C) and delta area (Figure 5D). Although without statistical significance (*p* = 0.12), the PRCC patients in the IS1 group manifested a declined survival probability relative to patients belonging to the IS2 group (Figure 5E). The principal component analysis (Figure 5F) indicated that the immunophenotyping of PRCC patients was robust, and the two subtypes were almost totally separated from each other. The proportion of immune subtypes in the age (<60 or >60), gender (male or female) and stage (I–IV) group were calculated and individually displayed (Figure 5G–I).

### 3.4. The Association of Immune Subtypes with Mutational Status

**Figure 6:**
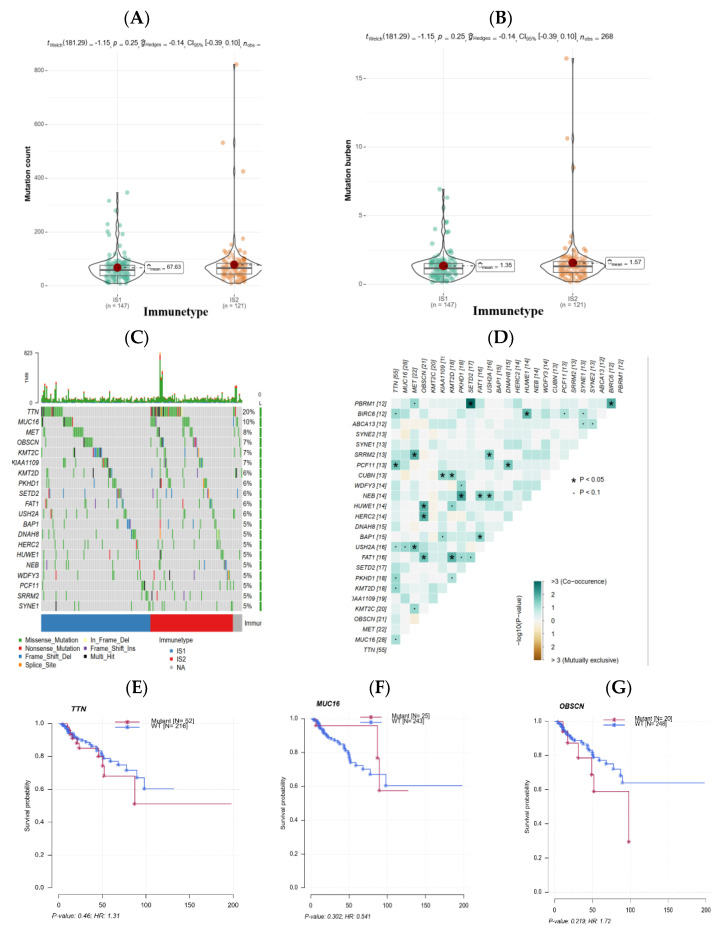
Mutation landscape of TCGA PRCC patients from different immune subtypes. (**A**,**B**) Mutation count and mutation burden evaluated in the two immune subtypes. (**C**) Top 20 genes in mutation frequency of the two subtypes. (**D**) The correlation of expression between most frequently mutated genes. (**E**–**G**) The survival diversity between the wild-type and mutant type of TTN, MUC16, and OBSCN.

Previously published studies have proved that, in most cases, higher mutation burdens of tumors (namely more mutation counts per million bases) possess relatively enhanced immunogenicity, ultimately impacting the effects of multiple immunological therapies including the mRNA vaccine. Given this, the count and burden of mutation in PRCC patients from TCGA were investigated and compared with the other immune subtype. As revealed in Figure 6A,B, related to IS1, IS2 had a trend of a higher mutation count as well as mutation burden. Subsequently, frequently mutated genes, such as TTN (20%) and MUC16 (10%), across two immune subtypes in PRCC patients were uncovered by somatic mutation analysis (Figure 6C), and the expression correlations among recurrent mutated genes were explored (Figure 6D). The impact of mutation in the three most common genes on patients’ survival was further analyzed, and results indicated that mutant types of TTN and OBSCN seemed to be poor indicators for prognosis (Figure 6E,G), however the wild-type of MUC16 was likely harmful to patients’ prognoses (Figure 6F). These findings disclosed that, with respect to the magnitude of mutation burden, there was a certain difference across immune subtypes and patients in IS2 may be more reactive to a mRNA vaccine.

### 3.5. Association between Immune Subtypes of PRCC and Immune Modulators

**Figure 7:**
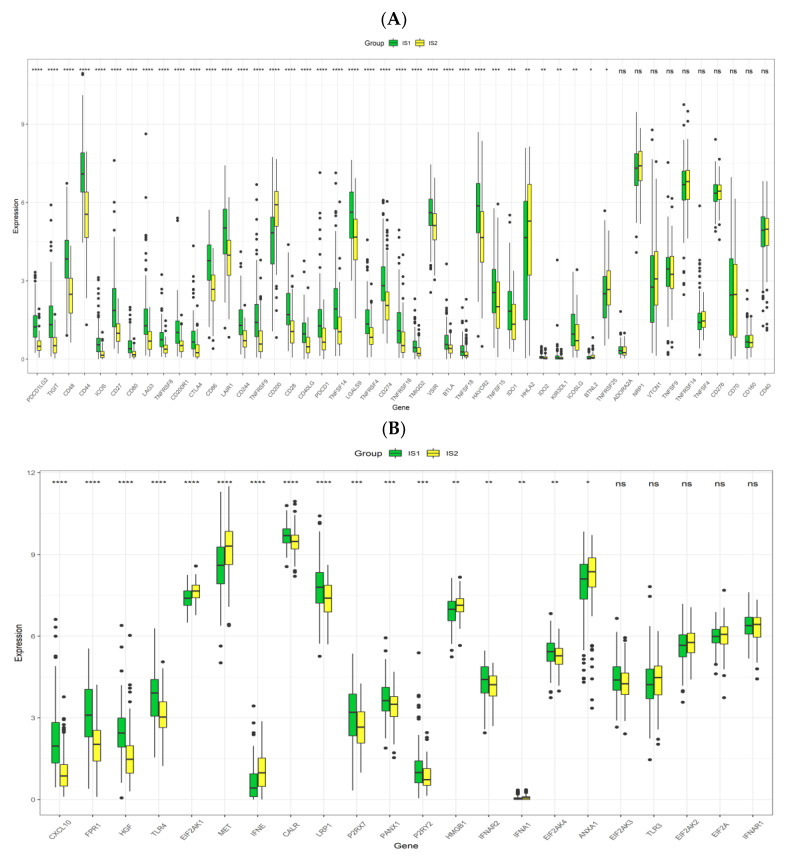
Expression associations between immune subtypes and ICPs and ICD modulators. (**A**) The expression pattern of ICPs among PRCC immune subtypes in the TCGA cohort. (**B**) Expression comparison of ICD modulators between the two subtypes in the TCGA cohort. * *p* < 0.01, ** *p* < 0.001, *** *p* < 0.0001, and **** *p* < 0.00001.

Immune modulators, especially inhibitory ICPs and stimulatory ICD modulators, play significant roles in the delicate control of immunity against cancer. In this context, the expression levels of these important players in the two groups were investigated. Among forty-seven ICPs (Figure 7A), differences in a total of thirty-seven genes were of statistical significance, and it was very clear that the vast majority (33 out of 37) of genes were more expressed in IS1, particularly PDCD1, CD274 and CTLA-4. Among twenty-two ICD-involved genes (Figure 7B), the differences between groups were noticeable in seventeen genes. There were eleven (CXCL10, FPR1, HGF, TLR4, CALR, LRP1, P2RX7, PANX1, P2RY2, IFNAR2, EIF2AK4) and six (EIF2AK1, MET, IFNE, HMGB1, IFNA1, ANXA1) genes with relatively higher expression in the IS1 and IS2 group, respectively. Therefore, immunotyping of patients was indicative of the expression levels of various ICPs and ICD modulators, and the effectiveness of a mRNA vaccine for IS1 patients could be undermined by these widely over-expressed ICPs in the microenvironment.

### 3.6. Cellular and Molecular Characteristics of Immune Subtypes

**Figure 8:**
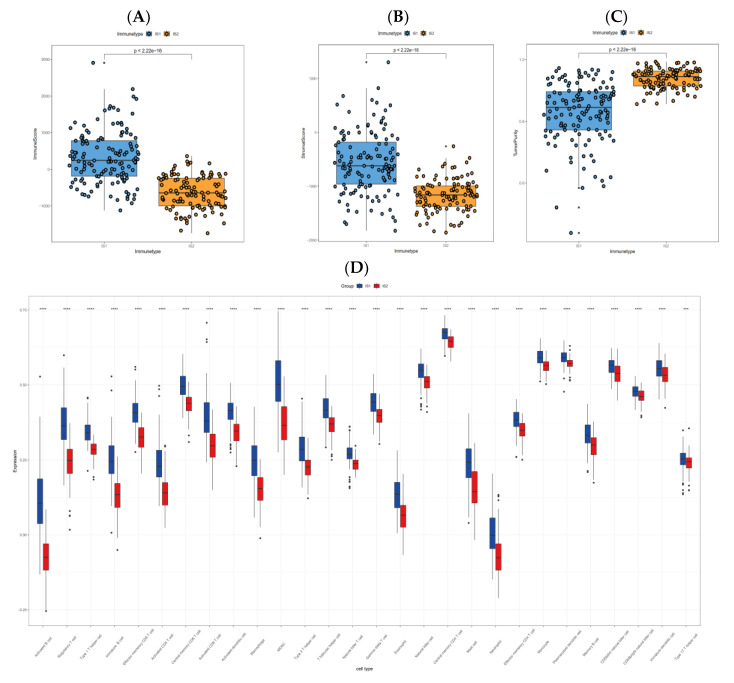

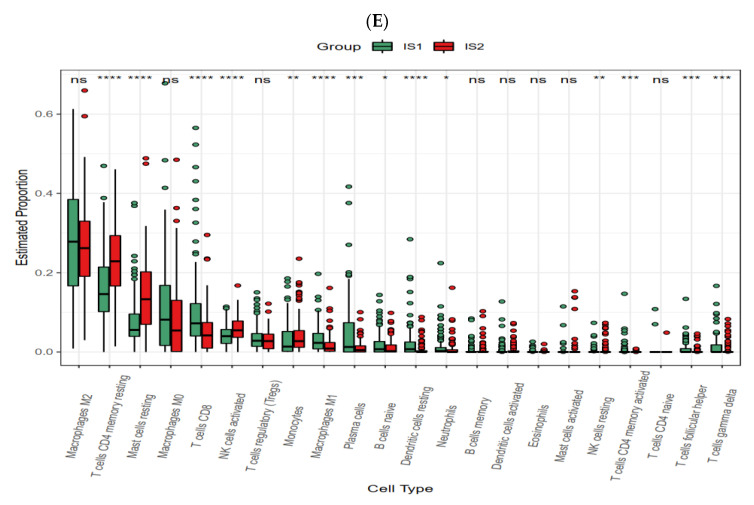
Analysis of the degree of immune cell infiltration in PRCC patients with different immune subtypes. (**A**–**C**) Immune score, stromal score and tumor purity of each sample were assessed by the ESTIMATE algorithm. (**D**) The immune cell infiltration pattern uncovered by the ssGSEA method. (**E**) Immune cell infiltration differences evaluated by CIBERSORT algorithm. * *p* < 0.01, ** *p* < 0.001, *** *p* < 0.0001, and **** *p* < 0.00001.

The gradually aggravated dysregulation of TIME is a pivotal hallmark of PRCC, inescapably disabling immune cells necessary for the clearance of malignant cells, which eventually leads to disease progression, even metastasis, and compromises the efficacy of many immunotherapies including the mRNA vaccine. At the beginning, revealed by the ESTIMATE algorithm, variations were grossly evident with the IS1 group possessing a higher immune score (Figure 8A), stromal score (Figure 8B) and lower tumor purity (Figure 8C), when compared with its counterpart. After that, ssGSEA assessing the richness of immune cells (in total, 28 kinds) was utilized to reveal differences between groups. Palpable distinctions, regarding the immune cell constitution, were demonstrated between the two subtypes (Figure 8D). On the whole, the IS1 group had more accumulation of activated B cells, activated CD4 T cells, activated CD8 T cells, natural killer cells, monocytes, type 1 T helper cells, myeloid-derived suppressor cells (MDSC), macrophages and regulatory T cells, among others. Lastly, the CIBERSORT analysis (Figure 8E) also exposed several remarkable dissimilarities. Compared to IS2, samples in IS1 exhibited larger proportions of some kinds of immune cells, for instance, the CD8 T cells, M1 macrophages, plasma cells, naïve B cells and resting dendritic cells; while samples in IS2 had larger proportions of activated NK cells, monocytes, resting CD4 memory T cells and resting mast cells.

### 3.7. Identification of Gene Co-Expression Modules of PRCC

**Figure 9:**
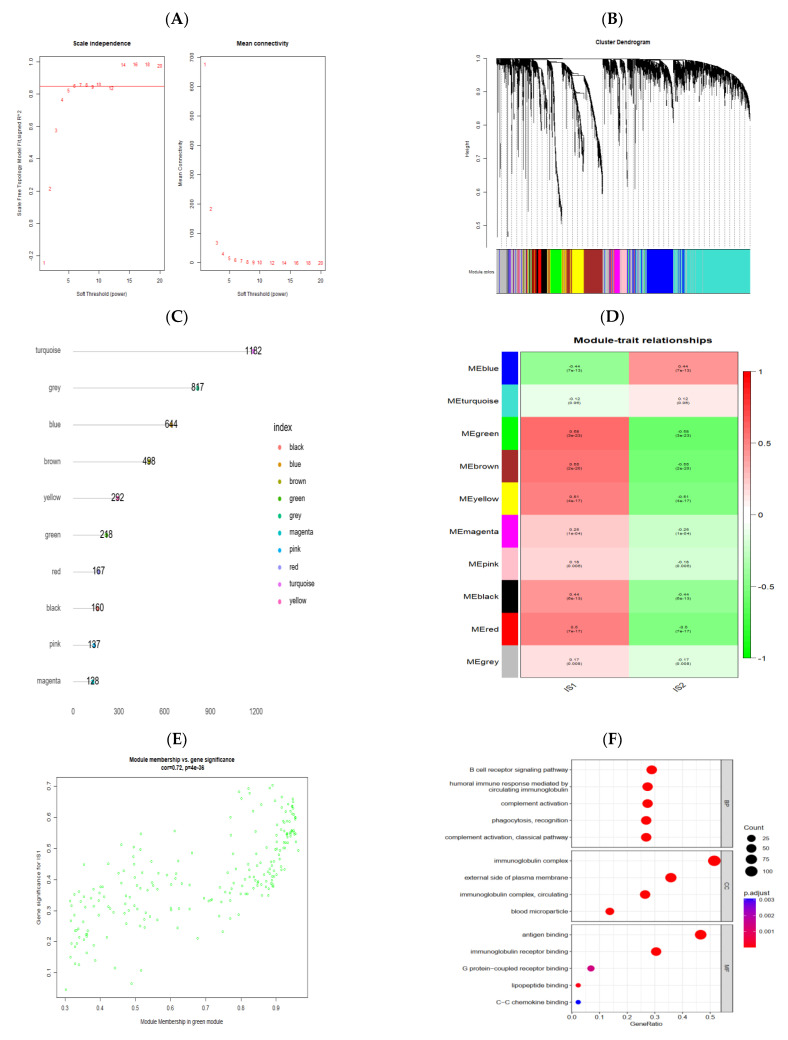
Identification of a gene co-expression network. (**A**) Scale-free fit index and mean connectivity under consecutive soft-thresholding powers (β). (**B**) Dendrogram of all differentially expressed genes clustered in accordance with a dissimilarity measure (1-TOM). (**C**) Dot plot of the co-expression gene modules. (**D**) Correlation analysis between modules and immune clusters. (**E**) Scatterplot of gene significance versus module membership to the green module. (**F**) GO functional enrichment analysis of genes in the green module.

Based on the expression similarity between any two highly variant genes, different modules were identified and further correlated with immune subtypes. To begin with, building on the results from mean connectivity combined with scale-free fit index, six was selected as the key parameter (soft-thresholding power) to enter into subsequent analyses (Figure 9A). Then, a total of ten modules with distinct colors were ascertained using average linkage hierarchical clustering (Figure 9B), and the number of genes involved in these modules were separately displayed (Figure 9C). Afterwards, the relationships between modules and traits (immune subtypes in this context) were explored, and for IS1, the three mostly positively correlated modules, judged by the magnitude of the correlation coefficient (Figure 9D), were the green module (MEgreen: rho: 0.58, *p* < 0.01), brown module (MEbrown: rho: 0.55, *p* < 0.01) and yellow module (MEyellow: rho: 0.51, *p* < 0.01), while blue module (MEblue: rho: −0.44, *p* < 0.01) was negatively correlated with IS1. Module membership versus gene significance analysis to the green module was performed and visualized by a scatter plot (Figure 9E) which, once again, confirmed the highly positive relationship between the green module and IS1 (r = 0.72, *p* < 0.01). Lastly, the GO enrichment analysis of genes extracted from the green module suggested that these genes primarily participated in the B cell receptor signaling pathway, humoral immune response, and phagocytosis, among others (Figure 9F).

### 3.8. Association between Immune Subtypes and Anti-Cancer Drug Sensitivity

**Figure 10:**
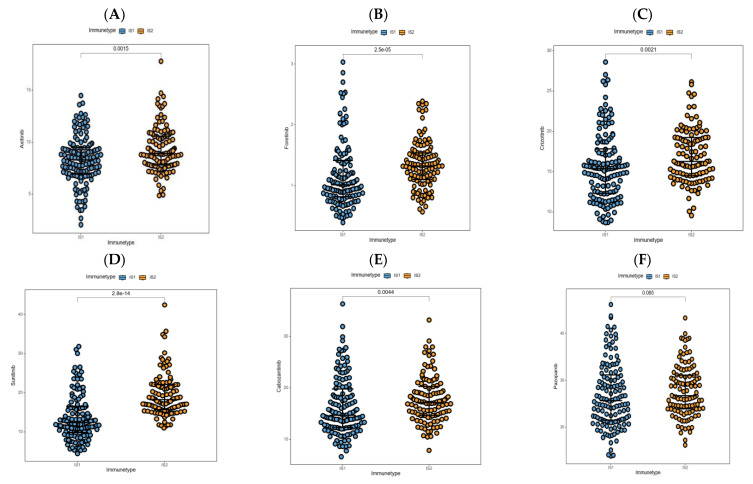
Anticancer drug analysis in PRCC patients with different immune types. (**A**–**F**) Half maximal inhibitory concentration (IC50) of multiple drugs in individuals with different immune types.

Drug sensitivity analysis of anti-cancer medicines available for PRCC patients was carried out in samples from the TCGA-PRCC cohort, which may provide some other options for patients who were less suitable for receipt of the mRNA vaccination. Surprisingly, for relatively lower IC50 values, most of these drugs were predicted to be more efficacious in patients clustered into IS1, including axitinib (Figure 10A), foretinib (Figure 10B), crizotinib (Figure 10C), sunitinib (Figure 10D), and cabozantinib (Figure 10E). In regard to pazopanib (Figure 10F), IS1 appeared to be more sensitive than IS2 (*p* = 0.085). Taken together, individuals in the IS1 group may receive a larger benefit from the targeted therapy, particularly drugs exploiting the vascular endothelial growth factor receptor (VEGFR) and MET proto-oncogene as targets, and these conspicuous divergences also confirmed the unavoidable heterogeneity among patients, reflecting the urgency of individualized treatments.

## 4. Discussion

In the last two decades, continuous and intensive efforts have been made by researchers across the world to develop drugs for patients with advanced renal cell carcinoma, and various medicines have been successively identified to improve the prognosis of these patients, notably those inhibiting the VEGF signal pathway [33]. As for the incidence rate, the papillary cell type is secondary to the clear cell type, accounting for about 50% of non-clear cell renal cell carcinoma, and the clinical management of this disease is similar to that of the clear type; for patients in early-stage, radical operation with maximal attempts to retain renal function is always given with a high priority, whereas molecular targeting of drugs is undoubtedly the basis of treatment for late-stage patients, since both types show little sensitivity to radiotherapy and chemotherapy [34,35]. Compared with the era of cytokines, obvious improvements in patients’ lifetimes have been obtained with VEGF receptor inhibitors; nevertheless, results from several clinical trials testing the efficacy of sunitinib or sorafenib suggest that the response rate and survival benefit of patients with PRCC are markedly inferior to that of patients with clear cell renal cell carcinoma, probably due to the disparate genetic background [36,37,38]. In addition, MET inhibitors and immune checkpoint blockers (cabozantinib and nivolumab, for instance) are also feasible for metastatic PRCC patients, displaying a higher responsivity than VEGF receptor inhibitors on the whole [39,40]. Despite these ameliorations, the overall clinical outcome of patients diagnosed with advanced PRCC is far from satisfactory, and most of them eventually die after suffering all kinds of treatment strategies, putting an insufferable financial burden on their families [41]. Hence, the exploration of new remedies is, without doubt, necessary to complement currently available drugs for the prolongation of survival time and the enhancement of quality of life in patients with PRCC.

Cancer vaccines, like immune checkpoint inhibitors and chimeric antigen receptor (CAR)-T cell immunotherapies, are also determined to reinvigorate the anti-tumor immunity of patients for the control of cancer and a longer survival period [42,43]. Dating back to 2006, the Gardasil quadrivalent vaccine has been approved by the Food and Drug Administration (FDA), and belongs to the prophylactic vaccines and indirectly prevents the oncogenesis of cervical cancer through the avoidance of high-risk human papillomavirus (HPV) infection [44]. Therapeutic cancer vaccines are more needed since most malignancies have no definite associations with viruses. In 2010, more excitingly, a therapeutic vaccine named Sipuleucel-T was approved by the FDA to treat prostate cancer patients at the hormone-refractory stage, and an increment (4.1 months) in median was observed when compared with the placebo group [45]. Cancer vaccines are mainly classified into cell-based (tumor/immune cells) and non-cell-based (peptide, DNA, mRNA) types [46]. The mRNA-type vaccine appears posterior to other types for several limitations such as instability, immunogenicity and impurities; however, after entering the 21st century, attributable to technological breakthroughs in optimizing the mRNA vaccine, it becomes more feasible than before [47]. The core of a mRNA vaccine is the mRNA sequence that is composed of two untranslated regions, one cap, one poly (A) tail and, more importantly, one open reading frame (ORF) encoding the vaccine antigen. Apart from the ORF, other structures are available to be modified, boosting the efficacy of mRNA vaccines. Exogeneous mRNA with innate immunogenicity can be recognized by endosomes and pattern recognition receptors, resulting in accelerated degradation or inhibitory translation of the mRNA; chemical modifications such as replacing cytidine and uridine with 5-methylcytidine and 5-methyluridine, respectively, can decrease the immunogenicity of mRNAs [48]. In addition, by increasing the GC proportion in the sequence, the stability of the mRNA can be enhanced, guaranteeing the expression effect of target antigens [49]. Currently, the most popular method used to synthesize mRNAs is in vitro transcription (IVT) which produces some unwanted sequences including short RNAs and double stranded RNAs, both of which disturb translation in vivo [50]. Other than decreasing Mg^2+^ concentration in the reaction mixture, recently, Baiersdorfer et al. has introduced a new method that can remove the vast majority of dsRNAs (up to 90%) [51]. Moreover, the mRNA-based vaccine exhibits several specific advantages compared to other vaccine types. First, it is characterized by delivering multiple tumor-associated antigens at a time and inducing the antibody-mediated humoral and cell-mediated immune responses synchronously, by which the possibility of vaccine resistance is decreased [52]. Second, unlike the peptide vaccine, it enables APCs to simultaneously present more epitopes via encoding whole-length tumor antigens, which can trigger a wider T cell response [53]. Last, it belongs to non-infectious vaccines and is free of proteins or contaminants derived from the production phase, thereby exhibiting better tolerability and safety than other types of vaccines [54]. Given the above-mentioned improvements and merits of mRNA-type vaccines, as well as a lack of vaccines in the clinic, it is worthwhile to develop such a vaccine for the treatment of PRCC patients.

In this study, somatic mutated and over-expressed genes in PRCC tissues were first disclosed as tentative tumor antigens. In order to pick out the genes with significant biological functions in PRCC, the prognostic value on overall survival and correlations with the abundance of APCs were further investigated. After that, five tumor antigens (ALOX15B, HS3ST2, PIGR, ZMYND15, and LIMK1) were identified to be candidates for the mRNA-based vaccine development, all of which were correlated with survival time of PRCC patients and the degree of APC infiltration. According to the above analyses, mRNA sequences of the five genes including ALOX15B, HS3ST2, PIGR, ZMYND15, and LIMK1 can be synthesized using the in vitro transcription (IVT) system and further modified by chemical methods. It was then encapsulated into lipid nanoparticles, and the optimized mRNA vaccine was injected into PRCC patients, with the aim of inducing immune responses against malignant cells. The potential of these tumor antigens for the development of an anti-PRCC mRNA vaccine has been proposed. Overexpression of HS3ST2 can increase the proliferation and colony-forming units of the BT-20 breast cancer cell line by enhancing the expression of anti-apoptotic molecules including survivin and XIAP [55]. The migratory and invasive ability of breast cancer MDA-MB-231 cells are significantly enhanced after the transfection of HS3ST2 [56]. Circulating extracellular vesicles from patients with advanced hepatocellular carcinoma have an abundance of PIGR, which can promote cancer aggressiveness together with stemness of Huh7 cells through activating the PDK1/Akt/GSK3β/β-catenin signaling axis, and PIGR is over-accumulated in hepatocellular cancer tissues predicting a decreased disease-free survival rate [57,58]. LIMK1 is highly expressed by colon cancer tissues, and inhibiting the expression of LIMK1 can reduce the proliferation, invasion and epithelial–mesenchymal transition of colon cancer cells by interacting with STK25 [59]. The expression of LIMK1 is significantly elevated in peritoneal metastatic tissues from patients with gastric cancer, and the knockout of LIMK1 can result in migration and invasion retardation of gastric cancer cells [60]. ALOX15B is highly enriched in colorectal cancer tissues in contrast to normal colorectal tissues and is also a poor indicator of patients’ overall survival [61].

Similar to other immunotherapeutic medicines for cancer, it was observed that only a portion of patients respond well to vaccine treatment and obtain extended survival time; in light of this, PRCC patients were classified into different groups with specific expression patterns of immune-associated genes to guide the use of mRNA-based vaccines in clinical practice. Two immune subtypes were finally identified, which exhibited diverse clinical and molecular characteristics. The IS1 group showed a trend of decreased overall survival possibilities (*p* = 0.12), and a larger sample size is needed to determine whether immunotypes could serve as a robust prognostic indicator for PRCC. Intriguingly, the two immune subtypes manifested significantly different sensitivities to several drugs that were routinely used in the clinical setting, additionally providing a reference for patients who were not suitable for vaccination and emphasizing the necessity of personalized treatment.

The efficacy of a mRNA-based cancer vaccine is greatly dependent on types of immune cells in the TIME and their functions. IS1 had a higher immune score than IS2, which reflected the immune “hot” and “cold” status of IS1 and IS2, respectively. The TIME of IS1 was rather complicated, and was composed of immune-stimulating immune cells such as activated CD8^+^ and CD4^+^ T cells, as well as immune-inhibiting immune cells, however these activated T cells were transformed to an exhausted phenotype as a result of overexpression of multiple immune-inhibitory molecules. Notably, expressions of regulatory T cells and myeloid-derived suppressor cells, which can inhibit the function of immune effector cells by various mechanisms [62], were significantly elevated in IS1. More importantly, the vast majority of ICP were highly expressed in the IS1, particularly the PD-1, PD-L1 and CTLA4, suggesting the immune-suppressive microenvironment in IS1, and this condition can largely weaken the performance of ICD modulators [63]. Taken together, IS1 was characterized by enhanced expressions of multiple immune-suppressive cells as well as molecules, both of which can compromise the efficacy of the mRNA vaccine, and PRCC patients in the IS1 can be administered with immune checkpoint blockers to improve the inhibitory status of TIME before using an anti-PRCC mRNA vaccine. Although the infiltration degree of immune cells in IS2 was lower than that in IS1, it still retained the expression of immune cells to some extent and receiving a mRNA vaccine in IS2 patients may trigger the hosts’ immune response towards a “hot” state to clear malignant cells.

## 5. Conclusions

In conclusion, ALOX15B, HS3ST2, PIGR, ZMYND15, and LIMK1 are potential targets for the development of PRCC mRNA vaccines, and patients who belong to the IS2 group may obtain more benefits from this therapy. Additionally, our research provides some insights into the design of an anti-PRCC mRNA vaccine and selects suitable patients to be vaccinated for future reference.

## Data Availability

All the original data are from public databases.
